# When the meningitis–encephalitis panel is negative: off-label joint infection PCR detects CTX-M ESBL–producing *Proteus mirabilis* in adult meningoencephalitis

**DOI:** 10.1186/s12879-026-13485-2

**Published:** 2026-05-04

**Authors:** Márk Kozák, Levente Majoros, Ferenc Bodnár, Rebeka Hodossy-Takács, Lilla Hudák, Lilla Rácz, Viktor Bencs, István Várkonyi

**Affiliations:** 1 Department of Infectology, Faculty of Medicine, University of Debrecen, 2-26 Bartok Bela Street, Debrecen, 4031 Hungary; 2 Division of Clinical Laboratory Sciences, Department of Laboratory Medicine, Faculty of Medicine, University of Debrecen, and MTA-DE Lendület “Momentum” Hemostasis and Stroke Research Group, Debrecen, Hungary; 3 Department of Neurology, Faculty of Medicine, University of Debrecen, Debrecen, Hungary

**Keywords:** *Proteus mirabilis*, Bacterial meningitis, Multiplex PCR, Off-label use, Extended-spectrum beta-lactamase

## Abstract

Spontaneous Gram-negative bacillary meningitis in adults is rare and carries high mortality, particularly in elderly patients with significant comorbidities. Although multiplex meningitis-encephalitis (ME) panels have improved diagnostic turnaround time, their limited pathogen spectrum may reduce sensitivity for certain clinically relevant Gram-negative organisms. We report a 78-year-old man presenting with acute meningoencephalitis, septic deterioration, and cerebrospinal fluid (CSF) findings strongly suggestive of bacterial infection. Despite marked neutrophilic pleocytosis and elevated protein levels, the multiplex ME panel was negative. Blood cultures subsequently yielded *Proteus mirabilis*. Given the discordance between inflammatory CSF findings and routine molecular testing, following interdisciplinary consultation, an off-label BioFire^®^ Joint Infection multiplex PCR panel was performed directly on CSF. The assay identified *Proteus mirabilis* harboring a cefotaximase-Munich (CTX-M)-type extended-spectrum β-lactamase (ESBL) resistance determinant. Based on this result, antimicrobial therapy was escalated from ceftriaxone-based empiric treatment to meropenem prior to availability of phenotypic susceptibility data. Despite early pathogen identification and targeted antimicrobial therapy, the patient died due to multiorgan dysfunction secondary to severe sepsis. This case suggests a potential diagnostic limitation of standard ME panels in selected high-risk scenarios and indicates that selective off-label use of broader multiplex PCR platforms may provide additional clinically relevant information in carefully selected patients. In the setting of high clinical suspicion and negative routine multiplex testing, rapid resistance-gene detection may provide actionable information within a decisive therapeutic window and support timely antimicrobial optimization in severe Gram-negative central nervous system infections. Further studies are required to evaluate the diagnostic performance and clinical impact of such approaches. Written informed consent for publication was obtained from the patient’s next of kin, and the study was conducted in accordance with institutional and ethical standards.

**Clinical trial number** Not applicable.

**Supplementary Information:**

The online version contains supplementary material available at 10.1186/s12879-026-13485-2.

## Introduction

Meningitis is a potentially life-threatening clinical syndrome defined as inflammation of the meninges. Bacterial meningitis is a worldwide health issue, especially in low-income countries where the incidence and the mortality rates are significantly higher than in high-income countries [1]. Typical clinical symptoms include fever, headache, neck stiffness and altered mental status. At least two of these clinical symptoms are present in approximately 95% of patients [2]. In addition, chronic neurological sequelae, like hearing loss, memory impairment, or focal neurologic deficits (e.g., hemiparesis) occur in approximately 9–25% of the cases, while overall mortality in adult bacterial meningitis remains substantial, ranging from approximately 10–30% despite appropriate antimicrobial therapy [3]. The causative pathogens of meningitis vary across patient populations. In community-acquired infections, the most frequent organisms include *Streptococcus pneumoniae (S. pneumoniae)*, *Haemophilus influenzae (H. influenzae)*, *Listeria monocytogenes (L. monocytogenes)*, *Neisseria meningitidis (N. meningitidis)*, and group B streptococci (GBS) [4]. Following the introduction of childhood vaccination programs, the incidence and mortality of bacterial meningitis have declined in high-income countries. Currently, *S. pneumoniae* remains the predominant causative organism, while GBS has emerged as an important pathogen in certain populations, and the incidence of *N. meningitidis* has declined substantially in recent years [4, 5].

In contrast, spontaneous meningitis caused by non-*H. influenzae* Gram-negative bacilli in adults is rare, most cases of Gram-negative bacillary meningitis (GNBM) occur in neonates and infants. Two clinical patterns have been described: post-neurosurgical or post-traumatic infections, and a spontaneous form occurring primarily in elderly patients with significant comorbidities such as diabetes, cirrhosis, malignancy, or recurrent urinary tract infections. GNBM in adults is associated with higher rates of neurological and systemic complications - including altered mental status, septic shock, and acute respiratory failure - as well as increased mortality compared with other etiologies of meningitis [3, 6]. In a large prospective single-center cohort study of spontaneous acute bacterial meningitis, non–*H. influenzae* Gram-negative bacilli accounted for approximately 9% of cases with established etiology, with an estimated annual incidence of about 2 cases per 100,000 adults [4]. Importantly, spontaneous GNBM carried a poor prognosis, with an in-hospital mortality of approximately 53% in that series, driven by both neurological and systemic complications [7]. In patients aged 80 years and older, atypical causative pathogens, including *Escherichia coli (E. coli)*, occur significantly more often than in younger adults (33% vs. 18%), while case fatality reaches 50% in this age group, highlighting the importance of considering Gram-negative pathogens in elderly patients with suspected bacterial meningitis [8]. In an earlier case series of 30 adult patients with Gram-negative bacillary meningitis reported by Berk and McCabe, *E. coli* was the most frequent pathogen, followed by *Klebsiella pneumoniae (K. pneumoniae)*, *Acinetobacter calcoaceticus*, *H. influenzae*, and *Pseudomonas aeruginosa (P. aeruginosa)*. Notably, *Proteus mirabilis (P. mirabilis)* was isolated in only one case, highlighting its rarity as a causative organism in adult meningitis, and underscoring the importance of reporting individual cases [4]. Consistent with these findings, recent case reports indicate that adult *P. mirabilis* meningitis remains an exceptionally rare entity, with only sporadic cases described in the literature [9–11].

*Proteus* spp. are opportunistic pathogens most frequently associated with urinary tract, wound, and intra-abdominal infections. Although they are part of the normal intestinal microbiota, central nervous system involvement is rare. When central nervous system infection occurs, it is typically secondary to bacteremia originating from a distant infectious focus, most commonly the urinary tract. The emergence of extended-spectrum beta-lactamase (ESBL)-producing strains has further complicated empirical treatment strategies, as these isolates may necessitate carbapenem therapy [6, 12].

Despite advances in rapid molecular diagnostics, pathogen identification in bacterial meningitis remains challenging in certain clinical scenarios. Cerebrospinal fluid (CSF) analysis remains the cornerstone of diagnosis of central nervous system infections. Typical findings in bacterial meningitis include neutrophilic pleocytosis, elevated protein concentration, and decreased CSF-to-serum glucose ratio. Early lumbar puncture is therefore recommended whenever no contraindication exists [13, 14]. Since 2015, multiplex polymerase chain reaction (PCR)–based meningitis–encephalitis (ME) panels have become widely available, allowing simultaneous detection of the most common bacterial, viral, and fungal pathogens within approximately one hour of sample processing. These panels typically detect common viral pathogens such as cytomegalovirus, enterovirus, herpes simplex virus 1 and 2, human herpesvirus 6, human parechovirus, and varicella zoster virus, as well as bacterial pathogens including *E. coli* K1, *H. influenzae*, *L. monocytogenes*, *N. meningitidis*, *S. agalactiae*, *S. pneumoniae*, and the fungal pathogen *Cryptococcus neoformans/gattii*. These platforms significantly shorten diagnostic turnaround time and support antimicrobial stewardship by facilitating earlier targeted therapy [15]. However, the pathogen spectrum of the standard ME panel is limited. Several clinically relevant Gram-negative bacilli, including *Proteus* spp., *Enterobacter* spp., *Serratia* spp., *Citrobacter* spp., and *P. aeruginosa* are not included among the detectable organisms representing a clinically relevant diagnostic gap, particularly in cases with discordant clinical and molecular findings. Consequently, a negative ME panel result does not exclude bacterial meningitis, particularly in elderly patients with multiple comorbidities and CSF findings highly suggestive of bacterial infection. In such high-risk populations, Gram-negative bacillary meningitis should remain in the differential diagnosis despite negative multiplex PCR results [7, 15].

In this context, broader multiplex PCR platforms originally developed for other specimen types, such as the BioFire^®^ Joint Infection PCR panel (bioMérieux, Marcy-l’Étoile, France), may provide additional diagnostic value. This assay detects a wide range of Gram-positive and Gram-negative pathogens and incorporates detection of selected antimicrobial resistance genes [16]. Although its use in CSF is considered off-label, emerging data suggest good concordance with culture results when applied to non-synovial samples, providing novel insights into pathogen identification and early resistance detection in critically ill patients [17].

Here, we report a case of adult meningoencephalitis in which the standard ME panel was negative, but off-label application of the Joint Infection PCR panel performed on CSF identified an ESBL-producing *P. mirabilis* harboring a cefotaximase-Munich (CTX-M)-type resistance gene, leading to early optimization of antimicrobial therapy. This case is uniquely valuable because it illustrates the combination of a discordant ME panel, off-label molecular diagnostics, and rapid resistance-gene detection that supported therapeutic decision-making.

## Case presentation

**Fig. 1:**
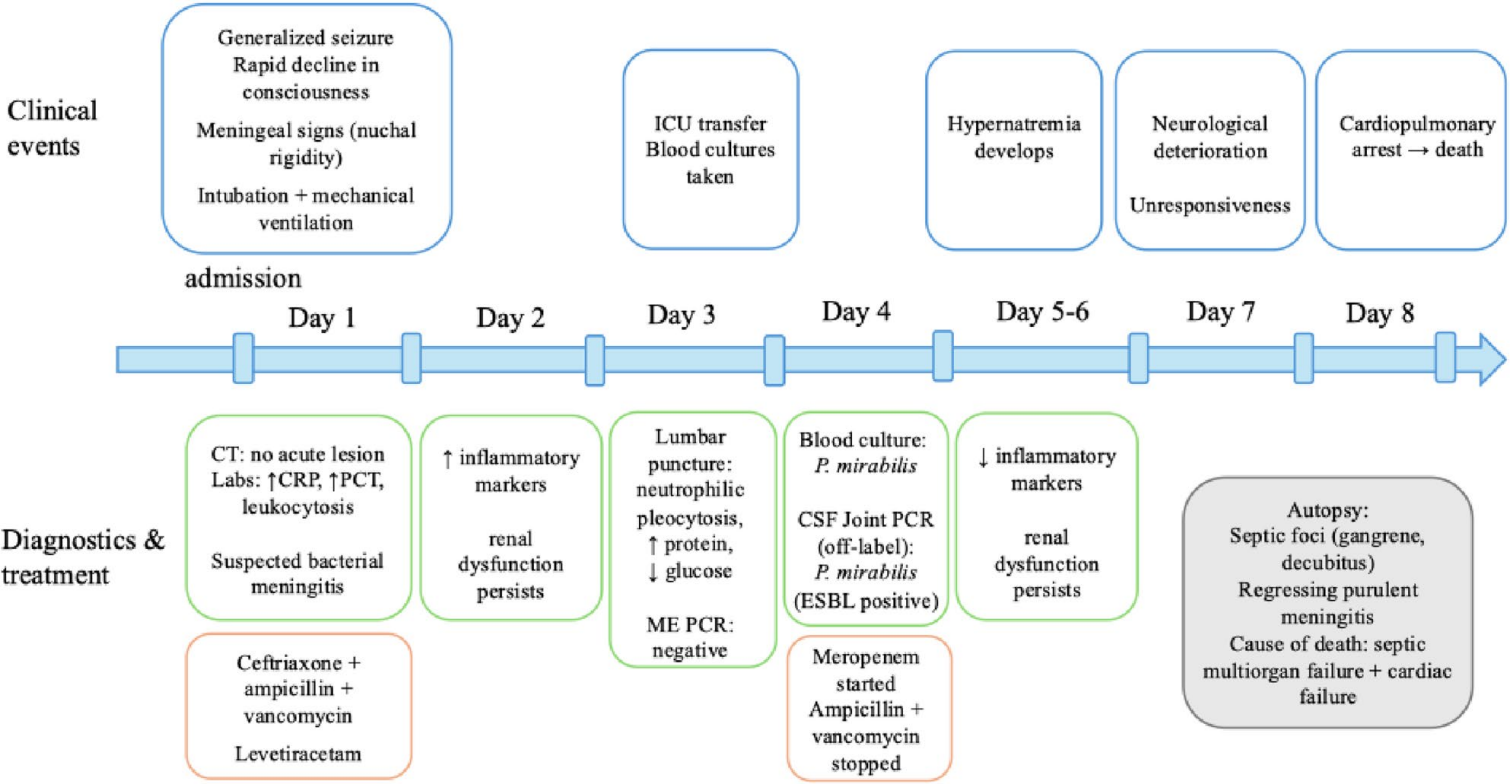
Clinical timeline of disease course and key diagnostic and therapeutic events. Timeline summarizing the patient’s clinical course, key diagnostic steps, microbiological findings, and antimicrobial therapy adjustments from presentation to outcome. Abbreviations: ME PCR, meningitis-encephalitis multiplex polymerase chain reaction; CRP, C-reactive protein; PCT, procalcitonin; CT, computed tomography; ICU, intensive care unit; P. mirabilis, Proteus mirabilis; CSF, cerebrospinal fluid; ESBL, extended-spectrum beta-lactamase

A 78-year-old man with a history of arterial hypertension, permanent atrial fibrillation on apixaban therapy, obesity, prior right hemispheric subacute ischemic stroke, chronic heart failure, chronic kidney disease, and advanced systemic atherosclerosis was admitted to the emergency department after a generalized tonic–clonic seizure followed by rapid deterioration of consciousness. According to family members, he had been neurologically stable approximately four hours prior to symptom onset. Due to impaired airway protection and persistent depressed consciousness, endotracheal intubation and mechanical ventilation were initiated upon arrival (day 1) with blood pressure 115/75 mmHg and heart rate 115/min. A timeline summarizing key clinical events, diagnostic steps, and therapeutic decisions is presented in Fig. 1.

On physical examination upon admission (day 1), multiple pressure ulcers were noted over the proximal lower extremities, and several toes showed signs of dry gangrene. Peripheral edema, ascites, and signs of chronic venous insufficiency were present. Neurological assessment revealed marked nuchal rigidity and conjugate gaze deviation to the left with sustained ocular fixation. Pupillary light reflexes were preserved, trigeminal nociceptive responses were symmetrical, and the gag reflex was intact. Corneal reflexes were absent bilaterally. Under sedation and mechanical ventilation, the Glasgow Coma Scale score was 3 (E1V1M1), although neurological assessment was limited by sedation.

Non-contrast cranial computed tomography (CT) showed no evidence of acute intracranial hemorrhage, mass effect, midline shift, or large territorial ischemia, and no signs of increased intracranial pressure (e.g., tonsillar herniation) were observed. Chronic structural abnormalities were present, including right-sided parietal (42 mm) and frontal (15 mm) encephalomalacic defects, as well as scattered periventricular and subcortical chronic vascular lesions and a lacunar infarct in the left lentiform nucleus.

Initial laboratory evaluation demonstrated marked inflammatory response with high-sensitivity C-reactive protein (hsCRP) (116 mg/L, reference < 5 mg/L) and procalcitonin (0.50 µg/L, reference < 0.05 µg/L), accompanied by leukocytosis (24.9 × 10⁹/L; 88% neutrophils). Renal dysfunction was present (urea 15.1 mmol/L, reference 2.8-8.0 mmol/L; creatinine 170 µmol/L, reference 62–106 µmol/L; estimated glomerular filtration rate (eGFR) 32 mL/min/1.73 m²), superimposed on baseline chronic kidney disease documented one month prior (urea 16.7 mmol/L, creatinine 157 µmol/L, eGFR 36 mL/min/1.73 m²), consistent with a component of acute kidney injury. Thrombocytosis (579 × 10⁹/L, reference 150–400 × 10⁹/L) and mild coagulopathy (initial prothrombin time (PT) 14.7 s, international normalized ratio (INR) 1.50; activated partial thromboplastin time (APTT) 35.6 s; thrombin time (TT) 17.1 s) were noted. Urinalysis demonstrated pyuria and bacteriuria, while nitrite testing was negative. Arterial blood gas analysis showed metabolic alkalosis with elevated bicarbonate levels (30.8–30.9 mmol/L, reference 22–31 mmol/L).

Abdominal ultrasonography demonstrated ascites, cirrhotic liver morphology, and cholelithiasis, consistent with the patient’s known chronic conditions. Chest imaging, including radiograph and non-contrast CT, revealed right-sided pleural effusion, basal dystelectasis, and cardiomegaly with coronary artery calcifications, but no evidence of acute parenchymal changes or pulmonary embolism. Overall, these findings represented chronic or previously documented abnormalities, with no new clinically significant thoracoabdominal or intracranial pathology detected.

Taken together, the presence of fever, marked inflammatory response, leukocytosis with neutrophil predominance, and pronounced meningeal signs (nuchal rigidity and impaired consciousness) raised a strong clinical suspicion of acute bacterial meningitis. Given the high clinical suspicion of community-acquired bacterial meningitis in an elderly patient, guideline-concordant empirical antimicrobial therapy was initiated immediately on day 1 according to current international guidelines (European Society of Clinical Microbiology and Infectious Diseases [ESCMID]) with intravenous ceftriaxone (2 g every 12 h), ampicillin (2 g every 4 h), and vancomycin (loading dose 25 mg/kg followed by 15 mg/kg every 12 h). This regimen was selected to provide broad coverage against the most common causative pathogens of community-acquired bacterial meningitis in elderly patients, including *S. pneumoniae*, *N. meningitidis*, and *L. monocytogenes*, while also addressing the possibility of penicillin-resistant pneumococci. Given the reduced estimated glomerular filtration rate (32 mL/min/1.73 m²), vancomycin dosing was adjusted on day 2 according to renal function and therapeutic drug monitoring, and ampicillin interval was modified simultaneously. Levetiracetam was initiated intravenously (750 mg twice daily) for seizure control, with dose adjustment according to renal function. On day 2, repeat laboratory assessment inflammatory markers further increased (CRP 158–167 mg/L; procalcitonin 3.69–3.85 µg/L), while leukocyte count decreased moderately (11.9 × 10⁹/L). Renal dysfunction persisted.

On day 3, after temporary discontinuation of apixaban and partial improvement in coagulation parameters (PT 13.2 s, INR 1.34; APTT 41.2 s; TT 17.1 s), lumbar puncture was safely performed. It should be noted that INR is not a reliable measure of anticoagulant effect for DOACs, and procedural decisions were guided by trends in coagulation tests, timing of the last apixaban dose, and interdisciplinary consultation. Cerebrospinal fluid analysis revealed marked neutrophilic pleocytosis (1,768 cells/µL; 87.8% polymorphonuclear leukocytes) with elevated protein concentration (total protein 632 mg/L, reference: 150–400 mg/L; albumin 348 mg/L, reference: 100–300 mg/L; IgG 71 mg/L, reference: 6.3–33.5 mg/L). CSF glucose was reduced relative to serum values (3.1 mmol/L versus 6.5 mmol/L, CSF/serum glucose ratio: 0.48). Multiplex ME PCR (BioFire^®^ FilmArray^®^ meningitis/encephalitis panel, bioMérieux, Marcy-l’Étoile, France) was negative. The patient was subsequently transferred to the Neurology Intensive Care Unit. Three pairs of blood cultures were obtained. Neurological examination under reduced sedation demonstrated persistent nuchal rigidity and somnolence.

Within 24 h, on day 4, two blood culture bottles yielded growth of *P. mirabilis*, identified by matrix-assisted laser desorption/ionization time-of-flight (MALDI-TOF) mass spectrometry. Samples were analyzed using a MALDI-TOF system (Bruker Microflex, Bruker Corporation, Billerica, MA, USA). Given the discrepancy between the negative multiplex ME panel and strong CSF inflammatory findings, further molecular testing was pursued after interdisciplinary discussion and approval by the treating clinical and microbiology teams, as no alternative confirmatory molecular assay was available in real time and the BioFire^®^ Joint Infection Panel was selected to provide rapid pathogen and resistance-gene detection in a critically ill patient. The CSF sample (approximately 3 mL) for off-label BioFire^®^ Joint Infection Panel PCR was processed immediately after collection under standard aseptic conditions, with internal controls confirming assay validity; however, the assay is not formally validated for CSF specimens and its use in this context is off-label, including at our institution, and results should be interpreted in the context of clinical and microbiological findings. The assay was performed according to the manufacturer’s instructions. No repeat testing was performed. Although strict sterile handling procedures were applied to minimize contamination risk. This panel is primarily validated for synovial fluid, however published data suggest overall concordance with conventional culture methods of approximately 85%, with a limit of detection around 10³–10⁴ colony-forming unit (CFU)/mL for bacterial targets [18]. The assay detected *P. mirabilis* harboring a CTX-M-type resistance gene. However, it should be noted, that the detection of CTX-M suggests the presence of an ESBL determinant but does not replace phenotypic susceptibility testing. Based on the molecular detection of a CTX-M–type resistance determinant and the patient’s critical clinical status, antimicrobial therapy was escalated to intravenous meropenem within 2 h to ensure effective coverage against a presumed ESBL-producing Gram-negative pathogen (initially 2 g every 8 h, subsequently dose-adjusted to 1 g every 12 h according to renal function). No further molecular characterization of the CTX-M enzyme subtype was carried out, and no confirmatory CTX-M PCR was performed on the blood isolate. Vancomycin and ampicillin were discontinued following pathogen identification and resistance profiling. Antimicrobial susceptibility testing of the isolated *P. mirabilis* demonstrated meropenem MIC of 0.064 µg/mL, confirming susceptibility.

Paracentesis performed for progressive ascites showed no evidence of spontaneous bacterial peritonitis. Surgical consultation for pressure ulcers and distal gangrene did not identify a need for urgent intervention.

On day 5–6, inflammatory markers declined following targeted therapy; however, renal function did not significantly improve, and hypernatremia developed during the ICU course (158 mmol/L).

Initial CSF cultures grew *Staphylococcus haemolyticus* and *Corynebacterium urealyticum*, likely contaminants based on the neutrophil-predominant CSF profile and early broad-spectrum therapy. Repeat CSF sampling on day 6 demonstrated growth of *S. haemolyticus*, *P. aeruginosa*, and *Proteus* spp.; in the context of prior antibiotics, these were interpreted cautiously. Given the concordant detection of *P. mirabilis* in blood cultures and by multiplex PCR, along with the marked inflammatory CSF profile, it was considered the most likely causative pathogen, though definitive confirmation of a single causative agent is not possible. The additional organisms were interpreted cautiously in the clinical context and were considered more consistent with contamination or transient colonization, although true polymicrobial infection cannot be fully excluded. Serum β-D-glucan and cryptococcal antigen testing were negative. Subsequent blood cultures yielded coagulase-negative staphylococci, considered contaminants. As CSF is a sterile compartment, the possibility of contamination or procedural factors cannot be excluded.

On hospital day seven, neurological status deteriorated further with progression to unresponsiveness. Renal function worsened, and oliguria required diuretic therapy. Despite maximal supportive care, the patient developed cardiopulmonary arrest on hospital day eight. Resuscitation efforts were unsuccessful.

Autopsy identified advanced hypertensive heart disease with eccentric left ventricular hypertrophy and arterio- and arteriolosclerotic nephrosclerosis. Multiple chronic ischemic and septic foci were present, including lower limb gangrene and a gluteal decubitus ulcer, which may have represented a potential source of infection leading to bacteremia and secondary central nervous system involvement. Purulent meningitis showed substantial regression under antimicrobial therapy. The immediate cause of death was determined to be acute cardiac failure in the context of multiorgan dysfunction secondary to severe sepsis.

## Discussion

Spontaneous meningitis caused by GNBM remains an uncommon clinical entity in adults and is most frequently described in association with neurosurgical procedures, head trauma, or other healthcare-associated exposures. Community-acquired cases are comparatively rare and typically occur in elderly patients with significant comorbidities, including chronic organ dysfunction, malignancy, or recurrent urinary tract infections. In this population, hematogenous dissemination from a distant infectious focus is considered the most likely mechanism of central nervous system (CNS) invasion [3, 6, 7].

Among Gram-negative organisms, *P. mirabilis* is only exceptionally implicated as a cause of adult meningitis, despite its well-established role in urinary tract and wound infections [6]. Previous observational cohorts have demonstrated that spontaneous GNBM in adults is associated with substantially higher rates of systemic complications - including septic shock and respiratory failure - and increased mortality when compared with meningitis caused by more common pathogens [7]. Accurate and timely pathogen identification is therefore essential, as delayed initiation of appropriate antimicrobial therapy may adversely influence neurological outcome and overall survival [2, 13].

In the present case, routine CSF analysis demonstrated a marked neutrophil-predominant inflammatory profile consistent with bacterial meningitis despite a negative multiplex ME panel result. This finding highlights an important diagnostic limitation of currently available ME PCR platforms, which are primarily designed to detect the most common community-acquired pathogens associated with acute CNS infections [19]. Available data indicate that the BioFire^®^ FilmArray^®^ ME panel demonstrates high diagnostic accuracy, with reported sensitivity of approximately 85–95% and specificity exceeding 95% compared with conventional reference methods. Reduced sensitivity has been reported for selected bacterial pathogens, particularly in partially treated infections [20]. In contrast, data on the diagnostic performance of the BioFire^®^ Joint Infection Panel in non-synovial specimens remain limited and performance characteristics may vary depending on specimen type, pathogen load, and prior antimicrobial exposure. It should also be noted that diagnostic performance may vary across different multiplex PCR platforms depending on assay design, target selection, and analytical sensitivity; therefore, findings related to a specific commercial system should not be generalized to all molecular diagnostic approaches. The BioFire^®^ FilmArray^®^ ME panel includes six bacterial targets; however, it does not encompass several Gram-negative bacilli implicated in spontaneous adult meningitis, including *Proteus* spp., *Enterobacter* spp., *Serratia* spp., and *P. aeruginosa* (Table 1) [19], highlighting an important diagnostic limitation in patients with suspected Gram-negative bacillary meningitis.

**Table 1:** Pathogens included in the BioFire^®^ FilmArray^®^ Meningitis/Encephalitis (ME) panel (bioMérieux, Marcy-l’Étoile, France) for cerebrospinal fluid analysis

Category	Target organism
Bacteria	*Streptococcus pneumoniae*
	*Neisseria meningitidis*
	*Haemophilus influenzae*
	*Listeria monocytogenes*
	*Streptococcus agalactiae*
	*Escherichia coli* K1
Viruses	Enterovirus
	Herpes simplex virus 1
	Herpes simplex virus 2
	Varicella-zoster virus
	Human herpesvirus 6
	Cytomegalovirus
	Human parechovirus
Fungi	*Cryptococcus neoformans/gattii*

In such clinical scenarios, the use of broader multiplex PCR platforms originally designed for other specimen types may provide additional diagnostic value. The BioFire^®^ Joint Infection Panel is a cartridge-based multiplex PCR assay with an approximate one-hour turnaround time that interrogates 39 microbial targets and nine antimicrobial resistance determinants, including CTX-M (Table 2) [16]. Although primarily validated for synovial fluid, its off-label application to non-synovial specimens has demonstrated high overall concordance with conventional culture methods (85.4%), overall accuracy of 89.6%, and excellent performance in CSF in a small subset of cases, with reported 100% concordance and accuracy [17]. Furthermore, the assay identified pathogens in 4.3% more cases than conventional culture methods and maintained good diagnostic performance in patients already receiving antimicrobial therapy at the time of sampling, a clinical situation in which culture-based detection may be compromised [17]. In addition, the panel demonstrated the ability to detect anaerobic pathogens that may be missed by conventional culture, further supporting the complementary role of multiplex PCR-based diagnostics in invasive infections [10]. These findings suggest that the BF-Joint Infection Panel may serve as an adjunct diagnostic tool in carefully selected high-risk patients when conventional CSF culture is expected to be slow or compromised, and when standard ME panels do not cover the suspected pathogen spectrum, highlighting its potential clinical benefit for rapid pathogen identification and early detection of antimicrobial resistance in severe CNS infections. Because nucleic-acid detection is less dependent on organism viability, BF-Joint Infection Panel maintained high performance in patients already receiving antibiotics at the time of sampling, underscoring its potential utility in partially treated infections where culture yield may be reduced [17].

However, off-label application of multiplex PCR assays to CSF carries potential limitations, including false-positive results due to contamination or detection of non-viable organisms, and false-negative results related to low pathogen load or limited panel coverage; therefore, results should be interpreted in the context of clinical and conventional microbiological findings [21]. Furthermore, the lack of formal validation for CSF specimens and potential matrix effects may influence assay performance, and findings should be interpreted with caution. Resistance-gene detection also requires careful interpretation, as molecular findings may not always be fully concordant with phenotypic susceptibility testing; however, in critically ill patients, rapid identification of clinically relevant resistance determinants may support earlier targeted antimicrobial escalation, which should subsequently be confirmed by culture-based identification and susceptibility testing interpreted according to the standards of the European Committee on Antimicrobial Susceptibility Testing (EUCAST) whenever feasible [17].

**Table 2:** Microbial targets and antimicrobial resistance genes included in the BioFire^®^ Joint Infection Panel (bioMérieux, Marcy-l’Étoile, France)

Category	Targets detected
Gram-positive bacteria	*Staphylococcus aureus*, *Staphylococcus lugdunensis*, *Streptococcus* spp., *Streptococcus agalactiae*, *Streptococcus pneumoniae*, *Streptococcus pyogenes*, *Enterococcus faecalis*, *Enterococcus faecium*, *Cutibacterium avidum/granulosum*, *Finegoldia magna*, *Parvimonas micra*, *Peptoniphilus* spp., *Peptostreptococcus anaerobius*, *Anaerococcus prevotii/vaginalis*, *Clostridium perfringens*
Gram-negative bacteria	*Escherichia coli*, *Haemophilus influenzae*, *Neisseria gonorrhoeae*, *Kingella kingae*, *Citrobacter* spp., *Enterobacter cloacae* complex, *Klebsiella aerogenes*, *Klebsiella pneumoniae* group, *Morganella morganii*, *Proteus* spp., *Pseudomonas aeruginosa*, *Salmonella* spp., *Serratia marcescens*, *Bacteroides fragilis*
Fungi	*Candida albicans*
Antimicrobial resistance genes	CTX-M, IMP, KPC, mecA/C and MREJ (MRSA), NDM, OXA-48-like, vanA/B, VIM

Abbreviations: CTX-M, cefotaximase-Munich; IMP, imipenemase; KPC, *Klebsiella pneumoniae* carbapenemase; mecA/C, methicillin resistance determinant; MREJ, methicillin-resistance junction; MRSA, methicillin-resistant *Staphylococcus aureus*; NDM, New Delhi metallo-β-lactamase; OXA-48-like, oxacillinase-48-like carbapenemase; vanA/B, vancomycin resistance determinant; VIM, Verona integron-encoded metallo-β-lactamase

In the present case, the off-label application of the BioFire^®^ Joint Infection Panel may have supported timely antimicrobial escalation. A detailed comparison of pathogen coverage between the ME panel and the Joint Infection panel is provided in Supplementary Table 1. The detection of a CTX-M resistance gene raised early suspicion of ESBL production, prompting timely escalation from ceftriaxone-based empirical therapy to meropenem. This is of particular importance, as infections caused by ESBL-producing Gram-negative organisms have been associated with worse clinical outcomes and increased mortality when effective antimicrobial therapy is delayed or inappropriate [22]. However, molecular detection of resistance genes does not replace phenotypic susceptibility testing and should be interpreted in conjunction with culture-based results. It should be noted that CTX-M enzymes account for the vast majority of ESBLs in Enterobacterales worldwide, their rapid identification is of high clinical relevance. The ability of multiplex PCR to detect resistance determinants within hours - compared with the days typically required for culture-based identification and phenotypic susceptibility testing - represents a substantial clinical advantage, particularly in critically ill patients [15, 17, 22]. Importantly, the clinical course in this case must be interpreted in the context of the patient’s advanced age, extensive comorbidities, and severe systemic illness at presentation. Consequently, while the use of off-label multiplex PCR directly influenced antimicrobial decision-making, its impact on clinical outcome cannot be established based on this one case. The patient was elderly with extensive comorbidities and presented with severe systemic illness. Therefore, the fatal outcome was most likely driven by severe sepsis, multiorgan dysfunction, and underlying chronic disease burden, and cannot be attributed to diagnostic delay or the applied diagnostic strategy.

To our best knowledge, similar cases have been rarely reported, and data on off-label application of joint infection PCR panels in CSF remain limited. Specifically, reports of spontaneous *P. mirabilis* meningitis with off-label application of a joint infection multiplex PCR panel enabling simultaneous pathogen identification and detection of a CTX-M resistance determinant in CSF are extremely limited. This observation may suggest a potential expansion of the diagnostic utility of rapid molecular platforms beyond their originally validated specimen types in carefully selected high-risk scenarios.

In conclusion, this case highlights the diagnostic limitations of standard ME panels in elderly high-risk patients with suspected bacterial meningitis. A negative multiplex result does not exclude Gram-negative bacillary infection when CSF findings are strongly suggestive of bacterial disease. Selective off-label application of broader multiplex PCR platforms incorporating antimicrobial resistance gene detection may provide potentially clinically actionable information in selected high-risk scenarios. Integration of rapid molecular diagnostics with conventional microbiology may be important to improve timely diagnosis and enable earlier targeted antimicrobial therapy in CNS infections. Earlier pathogen identification and resistance detection may facilitate timely optimization of antimicrobial therapy; however, a direct impact on clinical outcomes cannot be established based on single-case observations. Importantly, in this case, earlier antimicrobial escalation did not translate into a measurable improvement in clinical outcome. Further prospective studies are needed to validate the diagnostic performance and clinical impact of off-label multiplex PCR use in CSF.

Although this report demonstrates the potential clinical utility of off-label multiplex PCR testing in CSF, the findings should be considered hypothesis-generating, and several limitations must be acknowledged. First, conclusions are derived from a single case and therefore cannot establish generalizable diagnostic performance or outcome benefit. Second, the BioFire^®^ Joint Infection Panel is not formally validated for CSF specimens, and its analytical performance in this matrix requires further systematic evaluation. Importantly, the CSF multiplex findings were not independently confirmed by an orthogonal method and should therefore be interpreted with caution.Third, although early molecular detection of CTX-M supported antimicrobial escalation in this patient, definitive therapeutic decisions should continue to be guided by culture-based identification and standardized susceptibility testing interpreted according to the recommendations of the EUCAST whenever feasible. Finally, encephalitis as a distinct entity within the spectrum of meningoencephalitis cannot be definitively established based on the available data, and although the clinical presentation fulfills criteria for probable encephalitis according to the International Encephalitis Consortium, the lack of neuroimaging or electrophysiological confirmation limits diagnostic certainty.

## Electronic Supplementary Material

Below is the link to the electronic supplementary material.

Supplementary Material 1

## Data Availability

All relevant data supporting the findings of this case report are included within the article. Additional details are available from the corresponding author upon reasonable request, in accordance with institutional and ethical regulations.

## Abbreviations

APTT: Activated partial thromboplastin time
CFU: Colony-forming unit
CNS: Central nervous system
CSF: Cerebrospinal fluid
CTX-M: Cefotaximase-Munich
E. coli: *Escherichia coli*
eGFR: Estimated glomerular filtration rate
ESBL: Extended-spectrum β-lactamase
ESCMID: European Society of Clinical Microbiology and Infectious Diseases
EUCAST: European Committee on Antimicrobial Susceptibility Testing
GBS: Group B streptococci
GNBM: Gram-negative bacillary meningitis
H. influenzae: *Haemophilus influenzae*
hsCRP: High-sensitivity C-reactive protein
IMP: Imipenemase
INR: International normalized ratio
K. Pneumoniae: *Klebsiella pneumoniae*
KPC: *Klebsiella pneumoniae carbapenemase*
L. Monocytogenes: *Listeria monocytogenes*
MALDI-TOF: Matrix-assisted laser desorption/ionization time-of-flight
ME: Meningitis–encephalitis
mecA/C: Methicillin resistance determinant
MREJ: Methicillin-resistance junction
MRSA: Methicillin-resistant *Staphylococcus aureus*
N. meningitidis: *Neisseria meningitidis*
NDM: New Delhi metallo-β-lactamase
OXA-48-like: Oxacillinase-48-like carbapenemase
P. aeruginosa: *Pseudomonas aeruginosa*
P. mirabilis: *Proteus mirabilis*
PCR: Polymerase chain reaction
PT: Prothrombin time
S. pneumoniae: *Streptococcus pneumoniae*
TT: Thrombin time
vanA/B: Vancomycin resistance determinant
VIM: Verona integron-encoded metallo-β-lactamase
