# Supplementary material for: When the meningitis–encephalitis panel is negative: off-label joint infection PCR detects CTX-M ESBL–producing Proteus mirabilis in adult meningoencephalitis
Source: BMC Infect Dis. 2026 May 4;26:1186. doi: 10.1186/s12879-026-13485-2 (PMC13289471; doi:10.1186/s12879-026-13485-2)
Supplement: Supplementary file 1 — Supplementary Material 1 [file 12879_2026_13485_MOESM1_ESM.docx]

| **Category** | **ME Panel (Meningitis–Encephalitis PCR)** | **Joint Infection PCR Panel** |
| --- | --- | --- |
| **Viruses** | Cytomegalovirus; Enterovirus; Herpes simplex virus 1, 2; Human herpesvirus 6; Human parechovirus; Varicella zoster virus | Not included |
| **Gram-positive bacteria** | *Streptococcus agalactiae*; *Streptococcus pneumoniae* | *Staphylococcus aureus*; *Staphylococcus lugdunensis*; *Streptococcus spp.*; *Streptococcus agalactiae*; *Streptococcus pneumoniae*; *Streptococcus pyogenes*; *Enterococcus faecalis*; *Enterococcus faecium*; *Anaerococcus prevotii/vaginalis*; *Finegoldia magna*; *Parvimonas micra*; *Peptoniphilus*; *Peptostreptococcus anaerobius*; *Clostridium perfringens*; *Cutibacterium avidum/granulosum* |
| **Gram-negative bacteria** | *Escherichia coli* K1; *Haemophilus influenzae*; *Neisseria meningitidis* | *Escherichia coli*; *Haemophilus influenzae*; *Klebsiella pneumoniae* group; *Klebsiella aerogenes*; *Enterobacter cloacae* complex; *Citrobacter*; *Morganella morganii*; *Proteus spp.*; *Pseudomonas aeruginosa*; *Serratia marcescens*; *Salmonella spp.*; *Bacteroides fragilis*; *Neisseria gonorrhoeae*; *Kingella kingae* |
| **Other bacteria** | *Listeria monocytogenes* | Not included |
| **Fungi / Yeast** | *Cryptococcus neoformans/gattii* | *Candida spp.*; *Candida albicans* |

Supplementary Table 1. Comparison of pathogen spectra covered by the BioFire® FilmArray® meningitis–encephalitis (ME) panel and the BioFire® Joint Infection PCR panel (bioMérieux, Marcy-l’Étoile, France), highlighting the absence of certain Gram-negative bacilli (e.g., Proteus spp.) from the standard ME panel.
